# Supplementary figures and images for: Shift from visceral to subcutaneous adipose tissue in Cyp17a1-knockout rats prevents the progression of metabolic syndrome
Source: PLoS One. 2025 Dec 12;20(12):e0311478. doi: 10.1371/journal.pone.0311478 (PMC12700391; doi:10.1371/journal.pone.0311478)

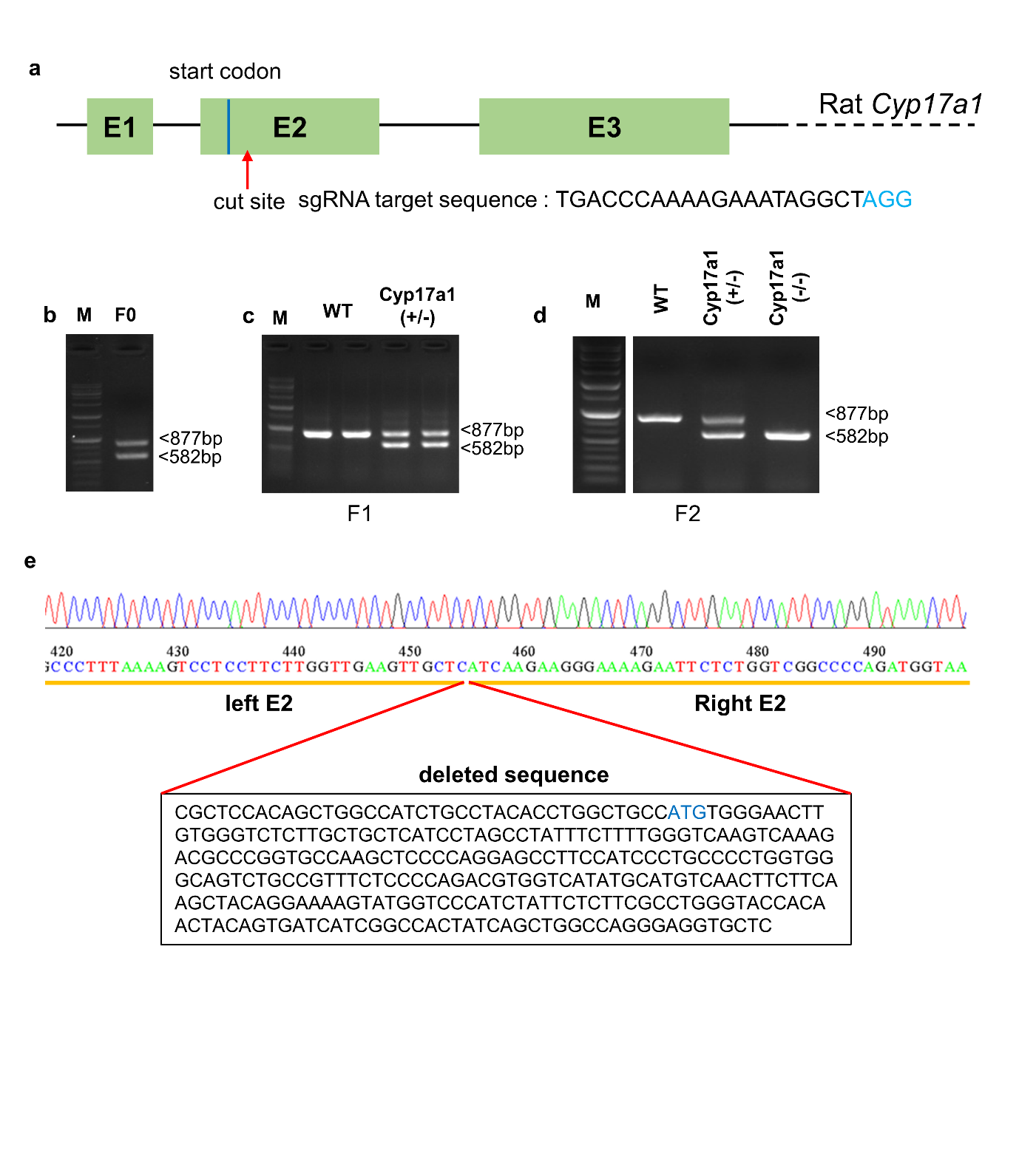


S1 Figure. Production of *Cyp17a1* knockout rats

Supplement: S1 Fig — a) Schematic image of CRISPR/Cas9 target site on rat Cyp17a1 gene. Blue line indicates start codon site and red arrow indicates cut site. b-d) PCR analysis results of F0, F1 and F2. F0 and Cyp17a1 (+/−) rats showed two bands, 877 bp and 582 bp, and wild-type rats and Cyp17a1 (−/−) rat showed one band, 877 bp upper band and 582 bp lower band each. e) Sanger sequencing result of Cyp17a1 knockout F0 rat. Box indicates deleted sequence, 295 bp. Blue letters, start codon. (DOCX) [file pone.0311478.s001.docx]

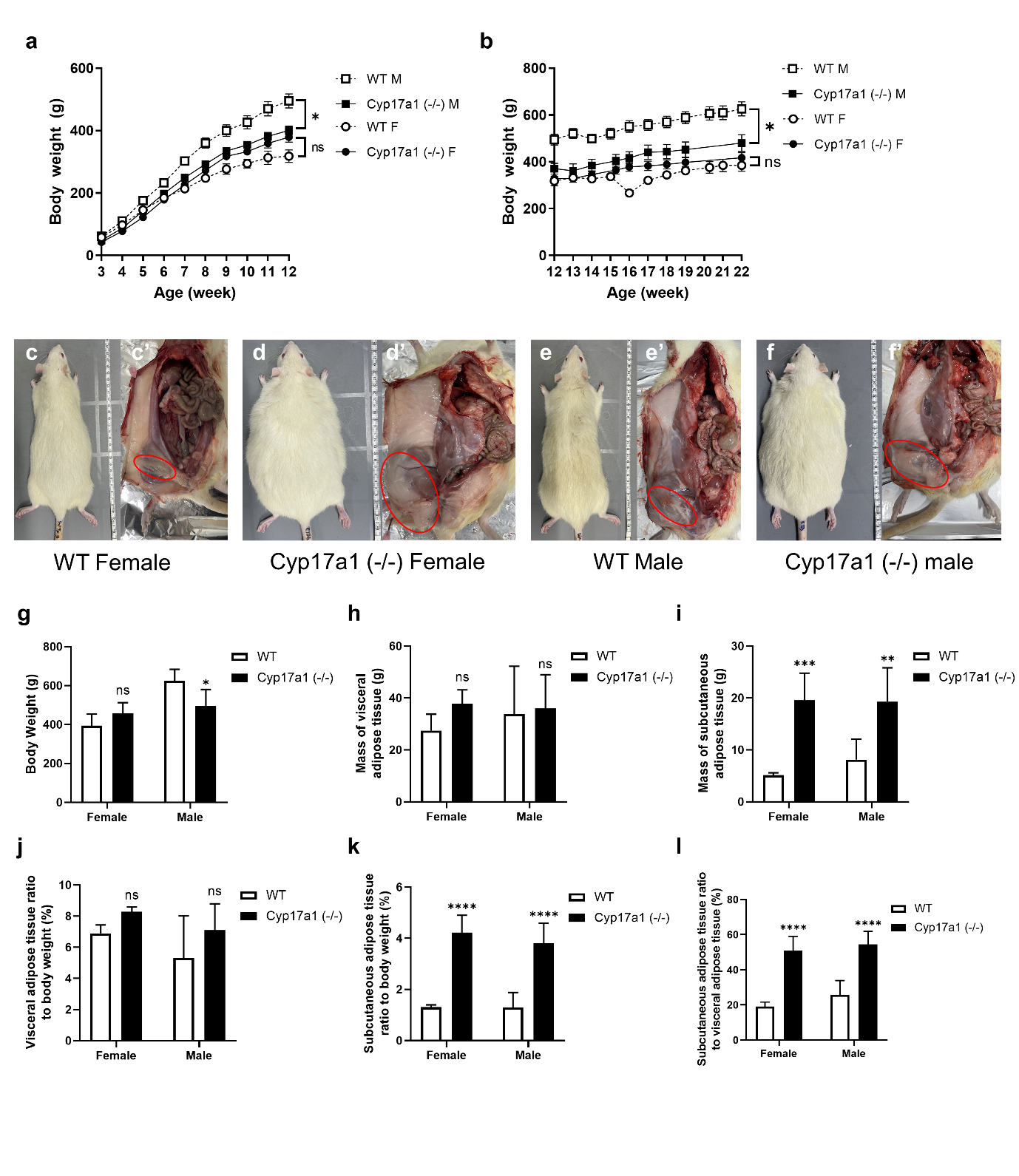


S2 Fig. Sampling results of chow diet fed *Cyp17a1* knockout rats

Supplement: S2 Fig — a) Body weight measurement of chow diet fed rats age from 3 to 12 week (n = 3). b) Body weight measurement of chow diet fed rats age from 12 to 20 week (n = 6, Cyp17a1 KO group; n = 3, wild-type group). c-f’) Representative images of subcutaneous adipose tissue of chow deit fed rats. Red circles indicates inguinal (subcutaneous) adipose tissue. g-l) Sampling results of rats. (g) Body weight, (h) mass of visceral adipose tissue, (i) subcutaneous adipose tissue, (j, k) relative mass of visceral and subcutaneous adipose tissue normalized to body weight. (l) Relative subcutaneous adipose tissue mass normalized to visceral adipose tissue (n = 9, Cyp17a1 KO male; n = 8, Cyp17a1 KO female, n = 3; wild-type group). ns, not significant, *p < 0.05, **p < 0.01, ***p < 0.001, ****p < 0.0001, higher than wild-type, using Student’s t-test. (DOCX) [file pone.0311478.s002.docx]

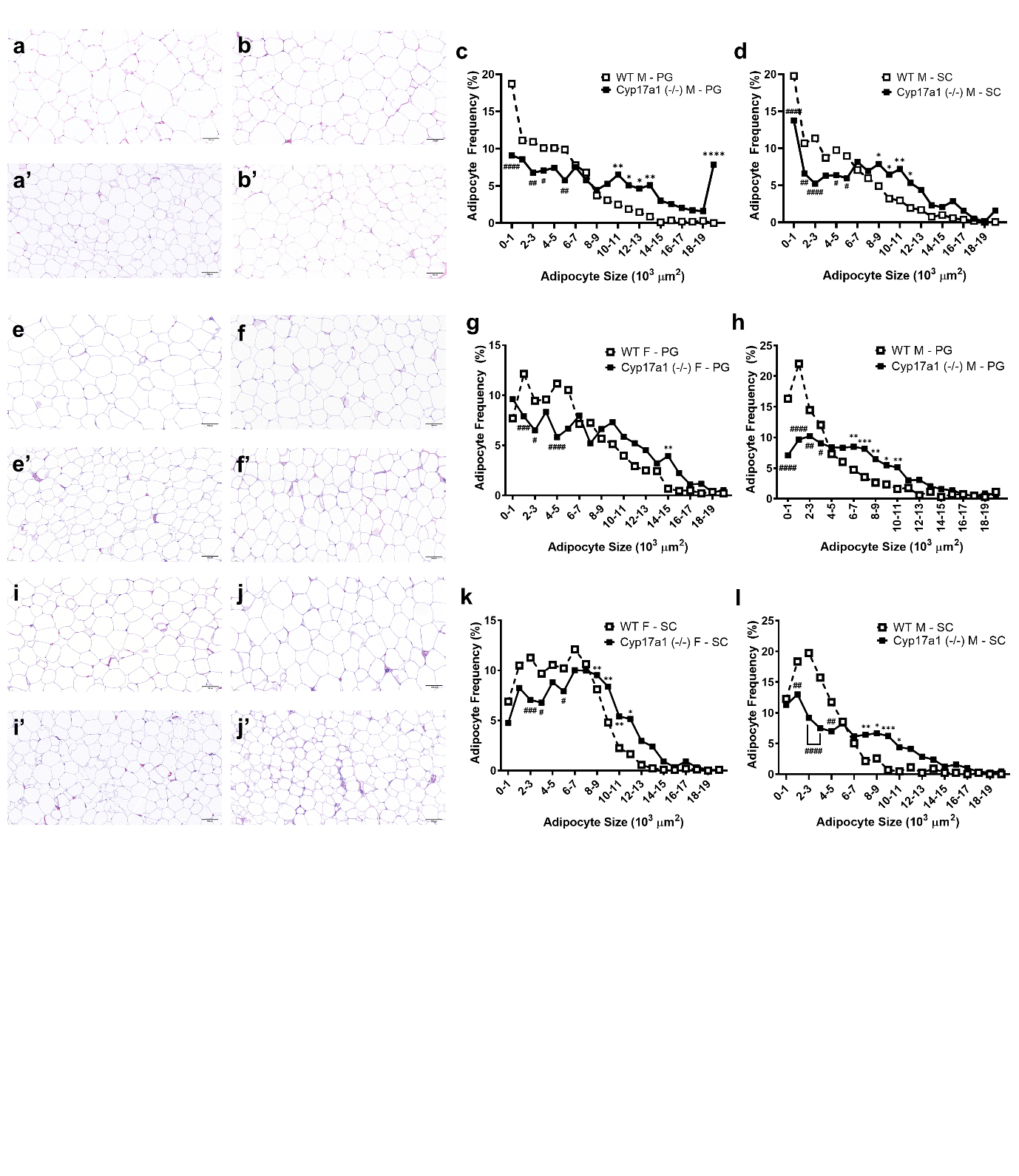


S3 Fig. Analysis of adipose tissue from *Cyp17a1* knockout rats

Supplement: S3 Fig — a, a’, b, b’) Representative images of H&E stained perigonadal adipose tissue (a, a') and subcutaneous adipose tissue (b, b’) of HFD fed male group. c, d) Adipocyte size analysis of perigonadal adipose tissue (c) and subcutaneous adipose tissue (d) from HFD fed male group. e, e’, f, f’) Representative images of H&E stained perigonadal adipose tissue (e, e') and subcutaneous adipose tissue (f, f’) of chow diet fed female group. g, h) Adipocyte size analysis of perigonadal adipose tissue (g) and subcutaneous adipose tissue (h) from chow diet fed female group. i, i’, j, j’) Representative images of H&E stained perigonadal adipose tissue (i, i') and subcutaneous adipose tissue (j, j’) of chow diet fed female group. k, l) Adipocyte size analysis of perigonadal adipose tissue (k) and subcutaneous adipose tissue (l) from chow diet fed female group. Scale bars = 100µm. #p < 0.05 ##p < 0.01, ###p < 0.001, #### p < 0.0001, lower than wild-type, *p < 0.05 **p < 0.01, ***p < 0.001, ****p < 0.0001, higher than wild-type, using Student’s t-test (n = 12). (DOCX) [file pone.0311478.s003.docx]

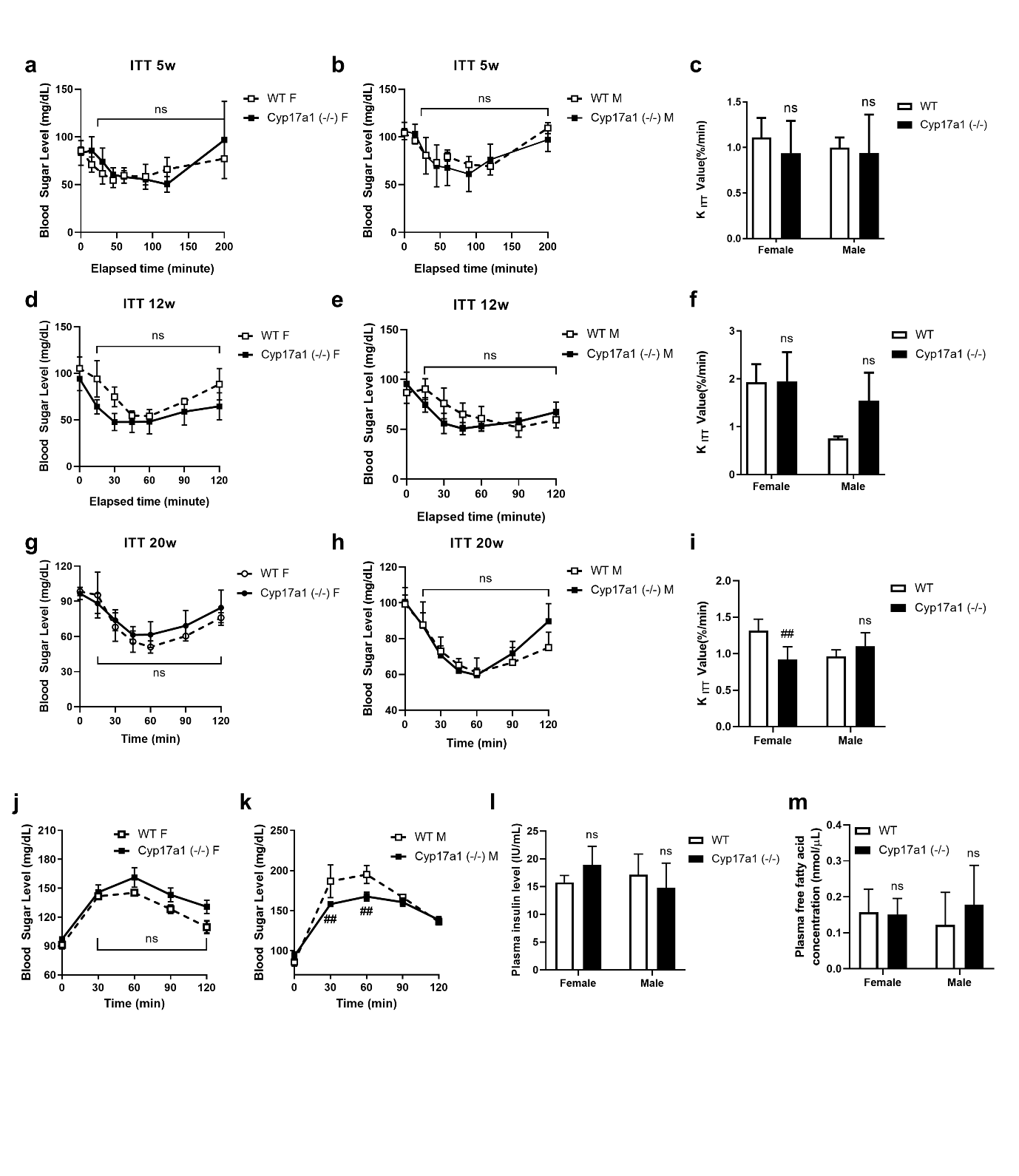


S4 Fig. Metabolic analysis in chow diet fed *Cyp17a1* knockout rats

Supplement: S4 Fig — a, d, g) Results of insulin tolerance test (ITT) of chow diet fed female rats at 5 week age (a), 12 week (d), 20 week (g) (n = 5, Cyp17a1 knockout females at 5 week, n = 6, Cyp17a1 knockout females at the other age, n = 3, wild-type females). b, e, h) Results of insulin tolerance test (ITT) of chow diet fed male rats at 5 week age (b), 12 week (e), 20 week (h) (n = 6, Cyp17a1 knockout males, n = 3, wild-type males). c, f, i) KITT value of chow diet fed rats at 5 week age (c), 12 week (f), 20 week (i). j, k) Results of oral glucose tolerance test (OGTT) of chow diet fed female (j) and male (k) rats. l) Plasma insulin level of chow diet fed rats analyzed with ELISA (n = 6, Cyp17a1 knockout group; n = 3 wild-type group). m) Plasma free fatty acid level of chow diet fed rats (n = 6, Cyp17a1 knockout group; n = 3 wild-type group). (DOCX) [file pone.0311478.s004.docx]

S1 Fig b, c

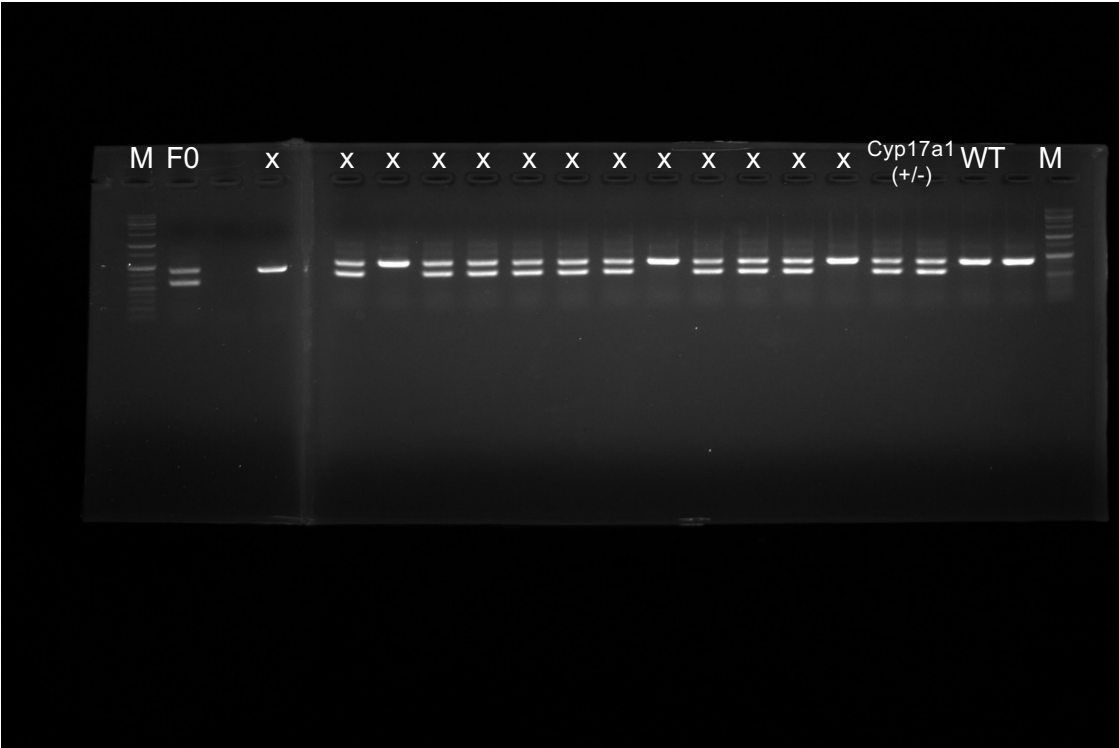

S1 Fig d

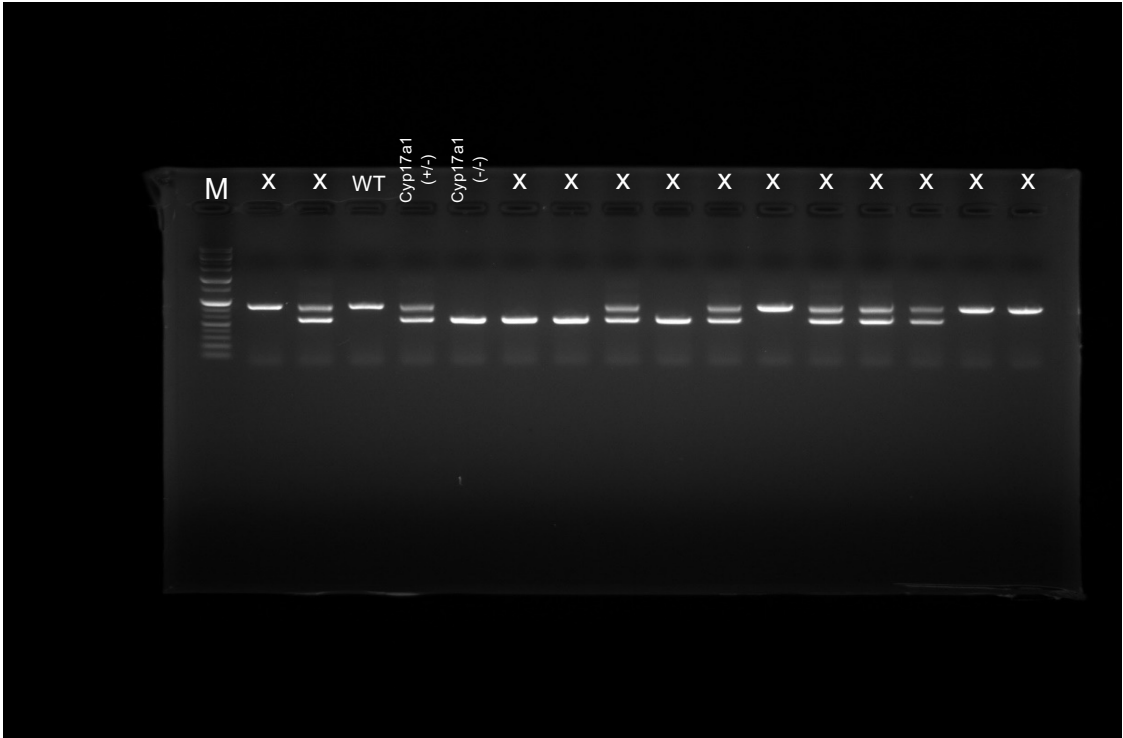

Supplement: S5 Fig — (PDF) [file pone.0311478.s005.pdf]
