# Supplementary material for: Shift from visceral to subcutaneous adipose tissue in Cyp17a1-knockout rats prevents the progression of metabolic syndrome
Source: PLoS One. 2025 Dec 12;20(12):e0311478. doi: 10.1371/journal.pone.0311478 (PMC12700391; doi:10.1371/journal.pone.0311478)
Supplement: S1 Table — (DOCX) [file pone.0311478.s006.docx]

| **Gene name** | **Forward Sequence (5’-3’)** | **Reverse Sequence (5’-3’)** |
| --- | --- | --- |
| Cyp17a1 - PCR | CTCTGTAACCCGATGGCAGT | CTCACCATCTTGCTGGACAA |
| Actb | GCTACAGCTTCACCACCACA | TCTCCAGGGAGGAAGAGGAT |
| Cyp17a1 | CGATGTGGGAACTTGTGGGT | TGCATATGACCACGTCTGGG |
| Klf5 | GAGACAGTGCCTCAGTGGTC | CCAGTTCTCAGGTGCGTGAT |
| Pparg | CGAGCTGGTATCTGCACTCA | AGCCCTCATCCTCCTGTTCT |
| Cebpa | TACCGAGTAGGGGGAGCAAA | CCTCTAAGGACAGGGACGGA |
| Srebf1 | ACAGGCTGAGAAAGGATGCT | TCAGTGCCAGGTTAGAAGCA |
| Dgat1 | TGAATTGGTGCGTGGTGATG | GACAGGCGCTTCTCAATCTG |
| Lipe | TCCAGTTCACACCTGCCATC | GTGCACGTCCAGGTTCTGTA |
| Prkaca | AGCTGTCCATCACCTCTTGC | GTCATATCCCCAGCAGCTCC |
| Pnpla | GACAGCTCCACCAACATCCA | AAGTCCATCTCGGTAGCCCT |
| Abhd5 | TGTGTATGCAGACCAGCAG | TTGGGTCTTGTCCGAGAACG |
| G0s2 | GCAGAGAAGCGAGACACAGA | GCAGCAAGTCAGTCCCAGAT |
| Mxipl | AGAGGACCCCAAACGCATTA | AGGGGCAAAGAGAGGGAATC |
| Slc2a4 | GCCGGACATTTGACCAGATC | CACCATTTTGCCCCTCAGTC |
| Acaca | CCACGCATACCCAGTTCAGT | GACACTGTTCACACGACCCT |
| Irs | TTCGGAGTCAGGGTTTCTGC | GCAGAGGGAGGTGTCGAAAA |
| Pik3ca | TGAACTGAGATGGCAGCTGG | TGTTCCTGACTGTGCCATCC |
| Il6 | TCCTTCCTACCCCAACTTCC | GGTTTGCCGAGTAGACCTCA |
| Tnf | AGATGTGGAACTGGCAGAGG | AGAAGAGGCTGAGGCACAGA |
| Agt | TCTTCTGCATCCTGACCTGG | TCTCGCAGGGTCTTCTCATC |
| Adipoq | TAAGGGTGACCCAGGAGATG | GGAACATTGGGGACAGTGAC |

S1 Table. Primer list used in PCR and qPCR analysis
