# Supplementary material for: Shift from visceral to subcutaneous adipose tissue in Cyp17a1-knockout rats prevents the progression of metabolic syndrome
Source: PLoS One. 2025 Dec 12;20(12):e0311478. doi: 10.1371/journal.pone.0311478 (PMC12700391; doi:10.1371/journal.pone.0311478)
Supplement: S2 Table — (DOCX) [file pone.0311478.s007.docx]

| **Blood Biochemistry Test**  **(mg/dL)** | **Male** | | | **Female** | | |
| --- | --- | --- | --- | --- | --- | --- |
|  | WT  (n=3)  (mean±SEM) | Cyp17a1 (-/-) (n=6)  (mean±SEM) | P-value  (WT-Cyp17a1) | WT  (n=3)  (mean±SEM) | Cyp17a1 (-/-) (n=6)  (mean±SEM) | P-value  (WT-Cyp17a1) |
| Triglyceride | 52±33.8 | 62±37.3 | 0.7177 | 13±12.7 | 50±23.8 | 0.0460 |
| Total Cholesterol | 54±9.0 | 54±15.9 | 0.9745 | 67±11.6 | 53±11.6 | 0.1313 |
| HDL | 19.5±3.04 | 23.1±7.74 | 0.4707 | 26.4±5.10 | 23.2±5.35 | 0.4201 |
| LDL | 6.0±1.16 | 6.2±1.62 | 0.8207 | 6.3±2.31 | 4.1±0.59 | 0.0481 |

S2 Table. Blood biochemistry of *Cyp17a1* knockout and wild-type rats with chow diet
